# PAI-1, MMP-9, and NLR combined with NIHSS for predicting 90-day poor functional outcome in elderly acute ischemic stroke: a prospective observational cohort study

**DOI:** 10.3389/fneur.2026.1793227

**Published:** 2026-04-15

**Authors:** Zilong Yang, Chunlan Hou, Baoai Wang

**Affiliations:** 1 Graduate School of Shanxi Medical University, Taiyuan, Shanxi, China; 2 Department of Neurology, Fenyang Hospital of Shanxi Province, Taiyuan, Shanxi, China

**Keywords:** acute ischemic stroke, MMP-9, NLR, PAI-1, predictive model

## Abstract

**Objective:**

To investigate whether a multi-marker panel comprising PAI-1, NLR, and MMP-9 enhances prognostication beyond the NIHSS score in elderly patients with acute ischemic stroke, and to develop a clinically applicable nomogram.

**Methods:**

A total of 113 elderly AIS patients and 63 elderly non-AIS controls were prospectively enrolled. Fasting venous blood samples were collected at 06:00 on the first morning after admission (or at 06:00 on the day of admission for overnight admissions), and serum PAI-1, MMP-9, and NLR were measured. Clinical data and NIHSS scores within 24 h of admission were collected. Outcomes were assessed at 90-day follow-up using the modified Rankin Scale (mRS) (favorable outcome: mRS ≤ 2; poor outcome: mRS > 2). Univariate and multivariate logistic regression analyses were performed to identify independent predictors. Three models were constructed: NIHSS alone, biomarkers alone, and their combination. Model performance was evaluated using ROC curves, calibration plots, decision curve analysis (DCA), and bootstrap internal validation.

**Results:**

Serum levels of PAI-1, MMP-9, and NLR were significantly higher in AIS patients than in controls (all *p* < 0.01). Among AIS patients, 50 (44.2%) had poor outcomes. In multivariable analysis, NIHSS score (OR, 2.24; 95% CI, 1.60–3.36; *p* < 0.001), MMP-9 (OR, 1.71; 95% CI, 1.32–2.35; *p* < 0.001), and NLR (OR, 1.42; 95% CI, 1.12–1.91; *p* = 0.011) were independently associated with poor outcome; PAI-1 showed a consistent effect direction but did not reach statistical significance (*p* = 0.077). The combined model achieved an AUC of 0.889 (95% CI, 0.831–0.946), significantly outperforming both the NIHSS-only model (AUC, 0.781; DeLong *p* = 0.003) and the biomarker-only model (AUC, 0.791; DeLong *p* = 0.005). The combined model demonstrated excellent calibration (Brier score 0.135), good internal validity (optimism-corrected C-index 0.874), and positive net benefit across a wide threshold probability range (0.15–0.80) on DCA.

**Conclusion:**

Elevated serum MMP-9 and NLR levels were independently associated with poor short-term prognosis in elderly AIS patients, while PAI-1 showed a consistent direction of association and contributed to the overall performance of the combined model. The nomogram combining these biomarkers with the NIHSS score provides improved risk stratification and may assist early clinical decision-making.

## 1 Introduction

Acute ischemic stroke (AIS) is a leading global cause of mortality and disability, characterized by high incidence and significant long-term impairment. Elderly AIS patients often present with poor vascular conditions and multiple comorbidities, leading to generally unfavorable prognoses and imposing substantial burdens on both families and society (1). Consequently, identifying sensitive indicators for disease assessment and accurate prognostic tools is of critical clinical importance for optimizing intervention strategies and improving patient outcomes. The pathophysiological process of AIS is complex, involving multiple key mechanisms such as fibrinolysis imbalance, systemic inflammatory response activation, and blood–brain barrier disruption. Plasminogen activator inhibitor-1 (PAI-1), a crucial negative regulator of the fibrinolytic system, impedes thrombolysis by inhibiting plasminogen activator activity, thereby exacerbating ischemic brain injury. Variations in PAI-1 levels have been demonstrated to correlate with the severity of cerebral infarction (2, 3). The neutrophil-to-lymphocyte ratio (NLR) serves as a convenient indicator reflecting systemic inflammation and immune balance. Post-ischemic inflammatory cascades and immunosuppressive states are often manifested as elevated NLR values. Given its ease of measurement and low cost, NLR is suitable for widespread use in primary healthcare settings (4, 5). Matrix metallopeptidase 9 (MMP-9) degrades vascular basement membranes and extracellular matrix components, compromising blood–brain barrier integrity and promoting cerebral edema and hemorrhagic transformation, thereby playing a key role in mediating secondary brain injury after ischemia (6). Although several studies have explored the association between individual biomarkers and prognosis in elderly AIS patients, limitations such as modest predictive power and insufficient specificity remain. Moreover, because a considerable proportion of elderly patients in routine clinical practice—particularly in regions with limited access to reperfusion therapy, where rural hospitals demonstrate significantly lower thrombolysis rates and worse functional outcomes compared with urban centers (7, 8)—are managed with standard medical treatment alone, there is a pressing need for accessible, blood-based prognostic tools that can facilitate early risk stratification in this underserved yet clinically important subgroup. Therefore, this study aims to analyze serum levels of PAI-1 and MMP-9, as well as NLR values, in elderly AIS patients who did not undergo reperfusion therapy, investigate their correlation with short-term prognosis, and develop a nomogram prediction model to support early risk stratification and personalized clinical management.

## 2 Materials and methods

### 2.1 Research subjects

**Figure 1:**
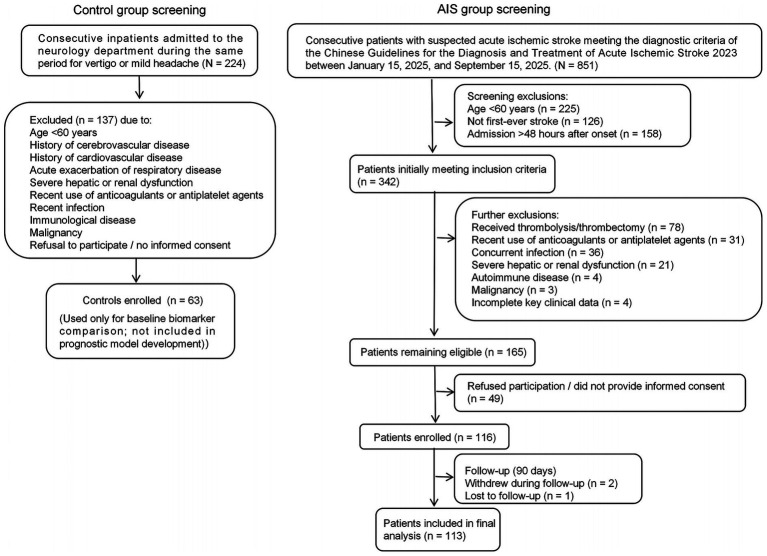
Flow diagram of patient screening and enrollment. The selection of non-AIS controls is shown on the left, and the flow of AIS patients is shown on the right. AIS, acute ischemic stroke. The control group was included for biomarker comparison only and was not used for prognostic model development.

A prospective cohort study was conducted. Between January 2025 and September 2025, consecutive patients with suspected acute ischemic stroke (AIS) admitted to the Department of Neurology, Fenyang Hospital, Shanxi Province, were screened for eligibility. Of 851 patients screened, 113 elderly patients with AIS were enrolled as the AIS group (see Figure 1 for detailed flow diagram).

The inclusion criteria were: (1) meeting the diagnostic criteria of the “Chinese Guidelines for the Diagnosis and Treatment of Acute Ischemic Stroke 2023” and confirmed by head CT or MRI (9); (2) first onset, age ≥ 60 years, hospitalized within 48 h of onset, and signed informed consent by the patient or their family; (3) provision of written informed consent by the patient or a legal representative.

The exclusion criteria were: (1) concomitant intracranial diseases (e.g., intracerebral hemorrhage, intracranial infection, or brain tumor); (2) History of recent systemic infection [defined as active infection at admission with body temperature ≥38.0 °C, white blood cell count >12.0 × 10^9^/L, or evidence of pneumonia, urinary tract infection, or other infectious diseases (10)]; (3) Severe hepatic or renal dysfunction [renal dysfunction defined as estimated glomerular filtration rate [eGFR] < 30 mL/min/1.73 m^2^ or requiring dialysis; hepatic dysfunction defined as alanine aminotransferase (ALT) > 3 × upper limit of normal or total bilirubin >2 × upper limit of normal (11)]; (4) History of autoimmune disease or malignancy; (5) Recent use of anticoagulant or antiplatelet agents; (6) Receipt of intravenous thrombolysis or mechanical thrombectomy during hospitalization. This criterion was prespecified to minimize treatment-related heterogeneity and confounding by indication, and to evaluate biomarker-associated prognostic signals in a relatively homogeneous non-reperfused cohort; (7) Inability to cooperate with the study or missing key clinical data.

During the same period, 63 patients admitted to the neurology department for vertigo or mild headache were enrolled as the control group (12, 13). The inclusion criteria for the control group were:(1) age ≥60 years; (2) absence of AIS and no history of cerebrovascular disease; (3) no recent history of infection; (4) absence of severe hepatic or renal dysfunction, cognitive impairment, hematological diseases, respiratory diseases, or cardiovascular diseases.

This study was conducted in accordance with the Declaration of Helsinki and was approved by the Ethics Committee of Fenyang Hospital, Shanxi Province on January 6, 2025 (Approval No. 2025061). All participants provided written informed consent prior to enrollment.

### 2.2 Specimen collection and index detection

According to our standardized protocol to ensure homogeneity of serum specimens, fasting cubital venous blood samples (5 mL) were collected from all participants at 6:00 a.m. on the morning following admission, or at 6:00 a.m. on the day of admission for those admitted overnight.

In accordance with clinical ethical requirements, all AIS patients received standardized treatment (statins, antiplatelet therapy, and fluid support) immediately after hospital admission. Consequently, blood sampling on the following morning occurred after several hours of treatment initiation. This timing reflects the real-world clinical reality that fasting blood samples cannot be obtained before treatment commencement in the acute stroke setting.

The blood was left to stand at room temperature for 30 min, then centrifuged at 3000 r/min at 4 °C for 15 min. The upper serum was separated and transferred to a sterile cryotube, numbered and stored at −80 °C for future testing. The levels of PAI-1 and MMP-9 in the serum were detected using ELISA kits (purchased from Quanzhou Ruixin Biotechnology Co., Ltd.), and the assays were performed strictly in accordance with the manufacturer’s instructions. At the same time, venous blood was collected for blood routine tests, and the neutrophil count and lymphocyte count were recorded. The NLR (NLR = neutrophil count/lymphocyte count) was calculated.

### 2.3 Clinical data collection and follow-up

General clinical data were collected for all enrolled patients, including sex, age, history of hypertension, history of diabetes, smoking history, and drinking history. Laboratory indices included triglycerides, total cholesterol, low-density lipoprotein cholesterol, high-density lipoprotein cholesterol, homocysteine, fasting blood glucose, glycated hemoglobin, D-dimer, and fibrinogen. The degree of neurological deficit was evaluated within 24 h of admission using the National Institutes of Health Stroke Scale (NIHSS). All data were collected at admission and entered into the study database concurrently.

Follow-up was scheduled at 30, 60, and 90 days after onset. All patients were informed of the follow-up plan and provided written informed consent before enrollment. Follow-up at 30 and 60 days was performed by telephone or outpatient visit depending on the patient’s condition. The final 90-day outcome assessment was conducted on-site in the outpatient clinic for all patients, and the modified Rankin Scale (mRS) was scored by a uniformly trained team of neurologists; each score was determined through discussion by at least two physicians, and any disagreement was adjudicated on site by a senior chief neurologist. The evaluators were blinded to baseline clinical data and biomarker results. A standardized mRS interview form was used for telephone follow-up to minimize assessment variability. One patient was lost to follow-up during the study period and was excluded from the final analysis (see Figure 1). Four patients with missing key clinical data (e.g., missing cranial MRI or hepatic/renal function indicators, making eligibility unverifiable) were excluded according to the predefined criteria.

### 2.4 Statistical methods

All analyses were performed in R (version 4.4.0). Two-sided *p* values < 0.05 were considered statistically significant.

#### 2.4.1 Descriptive statistics

Continuous variables were tested for normality using the Shapiro–Wilk test. Normally distributed variables are presented as mean ± standard deviation (SD) and compared using the Student’s *t*-test; non-normally distributed variables are presented as median with interquartile range (IQR) and compared using the Mann–Whitney U test. Categorical variables are expressed as counts (percentages) and compared using the *χ*^2^ test or Fisher’s exact test, as appropriate. Comparisons were performed for AIS versus non-AIS controls, and for AIS patients with favorable (mRS ≤ 2) versus poor outcomes (mRS > 2).

To account for baseline differences between AIS patients and controls, multivariate linear regression analyses were performed with each biomarker (PAI-1, MMP-9, NLR) as the dependent variable, group (AIS vs. control) as the independent variable of interest, and age, sex, smoking, drinking, diabetes, and hypertension as covariates. Results are reported as adjusted mean differences (*β*) with 95% confidence intervals (CI). Variance inflation factors (VIF) were calculated to assess multicollinearity.

Univariable logistic regression was conducted to identify candidate predictors of 90-day poor outcome. Results are reported as odds ratios (OR) with 95% confidence intervals (CI). Variables with *p* < 0.05 in univariable analysis were considered for multivariable modeling.

#### 2.4.2 Linearity and collinearity

The linearity assumption for continuous predictors (NIHSS score, PAI-1, MMP-9, NLR) was examined using restricted cubic splines with four knots placed at the 5th, 35th, 65th, and 95th percentiles. A *p* value for nonlinearity > 0.05 indicated that a linear relationship with the log odds of poor outcome was adequate. Multicollinearity among predictors was assessed using variance inflation factors (VIF), with VIF < 5 considered acceptable.

To evaluate the incremental predictive value of the biomarkers over NIHSS alone, three multivariable logistic regression models were constructed with 90-day poor outcome as the dependent variable: Model 1 (NIHSS only) included admission NIHSS score; Model 2 (biomarker only) included PAI-1, MMP-9, and NLR; Model 3 (combined) included all four variables.

#### 2.4.3 Model performance evaluation

Discrimination was quantified using the area under the receiver operating characteristic curve (AUC) with 95% CI calculated by the DeLong method. Pairwise comparisons of AUCs between models were performed using the DeLong test. Calibration was assessed graphically by plotting observed outcomes against predicted probabilities (calibration curve) and quantified by the calibration intercept and slope, as well as the Brier score (mean squared difference between predicted probabilities and observed outcomes); 95% CIs for Brier scores were obtained via bootstrap resampling (1,000 iterations). The Akaike information criterion (AIC) was used to compare model fit penalized for complexity.

To assess potential overfitting, we performed internal validation using bootstrap resampling with 1,000 iterations. The optimism-corrected C-index (equivalent to AUC for binary outcomes) was calculated for each model. The calibration curve was also bias-corrected using the same bootstrap procedure.

Clinical utility was evaluated using decision curve analysis (DCA), which estimates the net benefit of using the model to guide clinical decisions across a range of threshold probabilities. The net benefit of the combined model was compared with the “treat-all” and “treat-none” strategies, as well as with the two simpler models.

#### 2.4.4 Sensitivity analysis

To examine whether the timing of blood sampling influenced the results, we performed a sensitivity analysis by adding onset-to-blood draw time as an additional covariate to the combined model. Changes in regression coefficients, significance levels, AUC, Brier score, and AIC were evaluated.

#### 2.4.5 Missing data

During screening, four patients were excluded due to missing key clinical data (e.g., cranial MRI or liver/kidney function tests required to determine eligibility), and one patient was lost to follow-up during the 90-day period (Figure 1). All remaining 113 patients had complete data for all variables included in the analyses; therefore, no imputation was performed.

## 3 Results

### 3.1 Participant flow and study sample

A total of 851 consecutive patients with suspected acute ischemic stroke (AIS) were initially screened. Following the application of predefined inclusion and exclusion criteria, 113 AIS patients with complete clinical and laboratory data were included in the final prognostic analysis. Additionally, 63 age-eligible inpatients without a history of cerebrovascular disease were enrolled as a non-AIS control group; these subjects served solely as a reference for baseline biomarker comparison and were not included in the prognostic model development. A detailed flowchart of patient selection is provided in Figure 1.

### 3.2 Serum PAI-1, MMP-9, and NLR were significantly elevated in AIS patients

Baseline characteristics and biomarker levels for both groups are summarized in Table 1. Compared to the control group, AIS patients were older, had a higher proportion of males, and exhibited higher prevalences of smoking and hypertension. Serum concentrations of PAI-1, MMP-9, as well as the neutrophil-to-lymphocyte ratio (NLR), were significantly elevated in the AIS group (all *p* < 0.01). After adjusting for age, sex, smoking, drinking, diabetes, and hypertension in multivariable linear regression models, PAI-1, MMP-9, and NLR remained significantly elevated in AIS patients compared with controls (all *p* < 0.001; Supplementary Table S1).

**Table 1:** Baseline characteristics and biomarker levels in AIS patients and non-AIS controls.

Variable	Control (*n* = 63)	AIS (*n* = 113)	Statistic	*p* value
Demographics				
Age, years	66.00 (62.00, 68.50)	68.00 (65.00, 74.00)	*Z* = 3.54	0.001
Male, *n* (%)	31 (49.2)	81 (71.7)	*χ*^2^ = 7.89	0.005
Smoking, *n* (%)	8 (12.7)	49 (43.4)	*χ*^2^ = 15.99	<0.001
Drinking, *n* (%)	5 (7.9)	25 (22.1)	*χ*^2^ = 4.80	0.028
Diabetes, *n* (%)	11 (17.5)	31 (27.4)	*χ*^2^ = 1.70	0.192
Hypertension, *n* (%)	27 (42.9)	88 (77.9)	*χ*^2^ = 20.38	<0.001
Biomarkers				
NLR	2.51 (2.13, 3.19)	3.19 (2.22, 4.41)	*Z* = 3.03	0.002
PAI-1, ng/mL	5.76 (5.16, 7.45)	8.73 (6.90, 10.40)	*Z* = 7.07	<0.001
MMP-9, ng/mL	8.10 (7.71, 9.95)	10.38 (8.67, 12.12)	*Z* = 4.43	<0.001

Data are presented as mean ± SD, median (IQR), or *n* (%). Between-group comparisons were performed using the Student’s *t*-test (for normally distributed continuous variables) or Mann–Whitney U test (for non-normally distributed continuous variables), and the *χ*^2^ test or Fisher’s exact test for categorical variables, as appropriate. AIS, acute ischemic stroke; MMP-9, matrix metalloproteinase-9; NLR, neutrophil-to-lymphocyte ratio; PAI-1, plasminogen activator inhibitor-1.

### 3.3 Patients with poor outcome had higher NIHSS scores and biomarker levels

Among the 113 AIS patients, 63 (55.8%) achieved a favorable outcome (mRS ≤ 2) and 50 (44.2%) had a poor outcome (mRS > 2) at 90 days. As summarized in Table 2, patients with poor outcomes showed significantly higher admission NIHSS scores (median 4.0 vs. 2.0, *p* < 0.001) and higher D-dimer levels (median 0.60 vs. 0.50 mg/L, *p* = 0.025). Importantly, all three biomarkers were elevated in the poor-outcome group: PAI-1 (median 9.71 vs. 8.09 ng/mL, *p* = 0.001), NLR (median 3.58 vs. 3.03, *p* = 0.029), and MMP-9 (median 11.31 vs. 9.86 ng/mL, *p* < 0.001).

**Table 2:** Baseline clinical and laboratory characteristics of elderly AIS patients stratified by 90-day functional outcome.

Variable	Good outcome (*n* = 63)	Poor outcome (*n* = 50)	Statistic	*p* value
Demographics and history				
Age, years	68.00 (63.00, 73.00)	68.50 (67.00, 74.75)	*Z* = 1.30	0.192
Male, *n* (%)	46 (73.0)	35 (70.0)	*χ*^2^ = 0.02	0.886
Smoking, *n* (%)	28 (44.4)	21 (42.0)	*χ*^2^ = 0.01	0.945
Drinking, *n* (%)	13 (20.6)	12 (24.0)	*χ*^2^ = 0.04	0.842
Diabetes, *n* (%)	18 (28.6)	13 (26.0)	*χ*^2^ = 0.01	0.927
Hypertension, *n* (%)	52 (82.5)	36 (72.0)	*χ*^2^ = 1.24	0.266
Clinical characteristics				
SBP on day 2, mmHg	147.16 ± 18.45	144.86 ± 22.37	*t* = 0.59	0.560
Infarct size grade, *n* (%)			Fisher	0.250
≤ 1.5 cm	40 (63.5)	29 (58.0)		
1.5–5 cm	11 (17.5)	12 (24.0)		
5–10 cm	10 (15.9)	4 (8.0)		
≥ 10 cm	2 (3.2)	5 (10.0)		
Onset-to-sampling time, h	25.00 (11.50, 31.50)	21.50 (13.00, 33.75)	*Z* = 0.45	0.661
NIHSS score at admission	2.00 (1.00, 3.00)	4.00 (2.25, 6.00)	*Z* = 5.18	<0.001
TOAST, *n* (%)			Fisher	0.394
LAA	24 (38.1)	21 (42.0)		
SE	6 (9.5)	7 (14.0)		
SAO	32 (50.8)	19 (38.0)		
SOE	1 (1.6)	3 (6.0)		
Infarct location (anterior), *n* (%)	39 (61.9)	38 (76.0)	*χ*^2^ = 1.94	0.163
Leukoaraiosis, *n* (%)	31 (49.2)	25 (50.0)	*χ*^2^ = 0.00	1.000
Carotid plaque, *n* (%)	47 (74.6)	44 (88.0)	*χ*^2^ = 2.39	0.122
Laboratory markers				
Triglycerides, mmol/L	1.11 (0.88, 1.82)	1.13 (0.89, 1.60)	*Z* = 0.08	0.938
Total cholesterol, mmol/L	3.88 (3.29, 4.60)	4.04 (3.29, 4.78)	*Z* = 0.82	0.419
LDL-C, mmol/L	2.34 (1.83, 2.96)	2.58 (2.13, 3.05)	*Z* = 1.27	0.198
HDL-C, mmol/L	0.99 (0.85, 1.21)	0.99 (0.80, 1.25)	*Z* = 0.08	0.936
Homocysteine, μmol/L	16.90 (12.90, 23.05)	18.20 (13.48, 23.00)	*Z* = 0.44	0.658
Fasting blood glucose, mmol/L	5.20 (4.65, 6.20)	5.10 (4.61, 5.89)	*Z* = 0.49	0.629
HbA1c, %	5.90 (5.50, 6.75)	5.60 (5.32, 6.20)	*Z* = 1.42	0.160
D-dimer, mg/L	0.50 (0.43, 0.70)	0.60 (0.50, 1.15)	*Z* = 2.27	0.025
Fibrinogen, g/L	2.65 (2.21, 3.02)	2.68 (2.09, 3.25)	*Z* = 0.28	0.783
NLR	3.03 (2.07, 4.03)	3.58 (2.44, 6.06)	*Z* = 2.17	0.029
PAI-1, ng/mL	8.09 (6.43, 9.57)	9.71 (8.12, 11.20)	*Z* = 3.31	0.001
MMP-9, ng/mL	9.86 (7.93, 11.50)	11.31 (10.00, 12.64)	*Z* = 3.43	<0.001

Data are presented as mean ± SD, median (Q1, Q3), or *n* (%), as appropriate.

Between-group comparisons were performed using the Student’s *t*-test or Mann–Whitney U test for continuous variables and the *χ*^2^ test or Fisher’s exact test for categorical variables.

AIS, acute ischemic stroke; mRS, modified Rankin Scale; NIHSS, National Institutes of Health Stroke Scale; SBP, systolic blood pressure; LDL-C, low-density lipoprotein cholesterol; HDL-C, high-density lipoprotein cholesterol; NLR, neutrophil-to-lymphocyte ratio; PAI-1, plasminogen activator inhibitor-1; MMP-9, matrix metalloproteinase-9; HbA1c, glycated hemoglobin; TOAST, Trial of Org 10,172 in Acute Stroke Treatment; LAA, large artery atherosclerosis; SAO, small artery occlusion.

No significant between-group differences were observed in age, sex, smoking or drinking status, hypertension, diabetes, infarct location, leukoaraiosis, carotid plaque, fasting blood glucose, lipid profiles, homocysteine, HbA1c, or fibrinogen (all *p* > 0.05). Infarct size grade and onset-to-sampling time were also comparable between groups (*p* = 0.25 and *p* = 0.661, respectively).

### 3.4 Five variables were associated with poor outcome in univariable analysis

Univariable logistic regression identified five factors significantly associated with poor 90-day outcome: higher admission NIHSS score (OR, 1.86; 95% CI, 1.41–2.45; *p* < 0.001), elevated D-dimer (OR, 1.85; 95% CI, 1.02–3.38; *p* = 0.044), higher NLR (OR, 1.24; 95% CI, 1.05–1.48; *p* = 0.014), increased PAI-1 (OR, 1.39; 95% CI, 1.14–1.70; *p* = 0.001), and increased MMP-9 (OR, 1.42; 95% CI, 1.17–1.73; *p* = 0.001). Other demographic, clinical, and laboratory variables were not significantly associated with outcome (*p* > 0.05) (Table 3).

**Table 3:** Univariable logistic regression analysis for predictors of 90-day poor outcome.

Variable	OR (95% CI)	*p* value
Demographics and history		
Sex (male vs. female)	0.86 (0.38–1.96)	0.724
Age, per year	1.04 (0.98–1.11)	0.169
Smoking (yes vs. no)	0.91 (0.43–1.92)	0.795
Diabetes (yes vs. no)	0.88 (0.38–2.03)	0.761
Hypertension (yes vs. no)	0.54 (0.22–1.33)	0.183
Clinical characteristics		
SBP on day 2, per mmHg	0.99 (0.98–1.01)	0.547
Infarct size grade		
≤ 1.5 cm	1.00 (ref)	
1.5–5 cm	1.50 (0.58–3.88)	0.398
5–10 cm	0.55 (0.16–1.93)	0.353
≥ 10 cm	3.45 (0.62–19.03)	0.155
Onset-to-sampling time, per hour	1.01 (0.98–1.04)	0.674
NIHSS score at admission, per point	1.86 (1.41–2.45)	<0.001
TOAST		
LAA	1.00 (ref)	
SE	1.33 (0.384–4.75)	0.649
SAO	0.679 (0.298–1.53)	0.351
SOE	3.43 (0.404–72.3)	0.302
Infarct location (anterior vs. posterior)	1.95 (0.85–4.44)	0.113
Leukoaraiosis (yes vs. no)	1.03 (0.49–2.17)	0.933
Carotid plaque (yes vs. no)	2.50 (0.90–6.95)	0.080
Laboratory markers		
Triglycerides, per mmol/L	0.99 (0.61–1.60)	0.961
Total cholesterol, per mmol/L	1.23 (0.85–1.79)	0.275
LDL-C, per mmol/L	1.40 (0.90–2.18)	0.132
HDL-C, per mmol/L	0.82 (0.46–1.47)	0.512
Homocysteine, per μmol/L	0.99 (0.98–1.01)	0.619
Fasting blood glucose, per mmol/L	0.94 (0.78–1.12)	0.471
HbA1c, per %	0.79 (0.59–1.07)	0.130
D-dimer, per mg/L	1.85 (1.02–3.38)	0.044
Fibrinogen, per g/L	1.19 (0.81–1.74)	0.371
NLR	1.24 (1.05–1.48)	0.014
PAI-1, per ng/mL	1.39 (1.14–1.70)	0.001
MMP-9, per ng/mL	1.42 (1.17–1.73)	0.001

All estimates were derived from univariable logistic regression models with poor outcome (mRS > 2) as the dependent variable.

For categorical variables, the reference group is indicated in parentheses.

OR, odds ratio; CI, confidence interval; NIHSS, National Institutes of Health Stroke Scale; SBP, systolic blood pressure; LDL-C, low-density lipoprotein cholesterol; HDL-C, high-density lipoprotein cholesterol; HbA1c, glycated hemoglobin; NLR, neutrophil-to-lymphocyte ratio; PAI-1, plasminogen activator inhibitor-1; MMP-9, matrix metalloproteinase-9; TOAST, Trial of Org 10,172 in Acute Stroke Treatment; LAA, large artery atherosclerosis; SAO, small artery occlusion.

### 3.5 All continuous predictors exhibited linear relationships with log odds (P for nonlinearity > 0.05)

**Figure 2:**
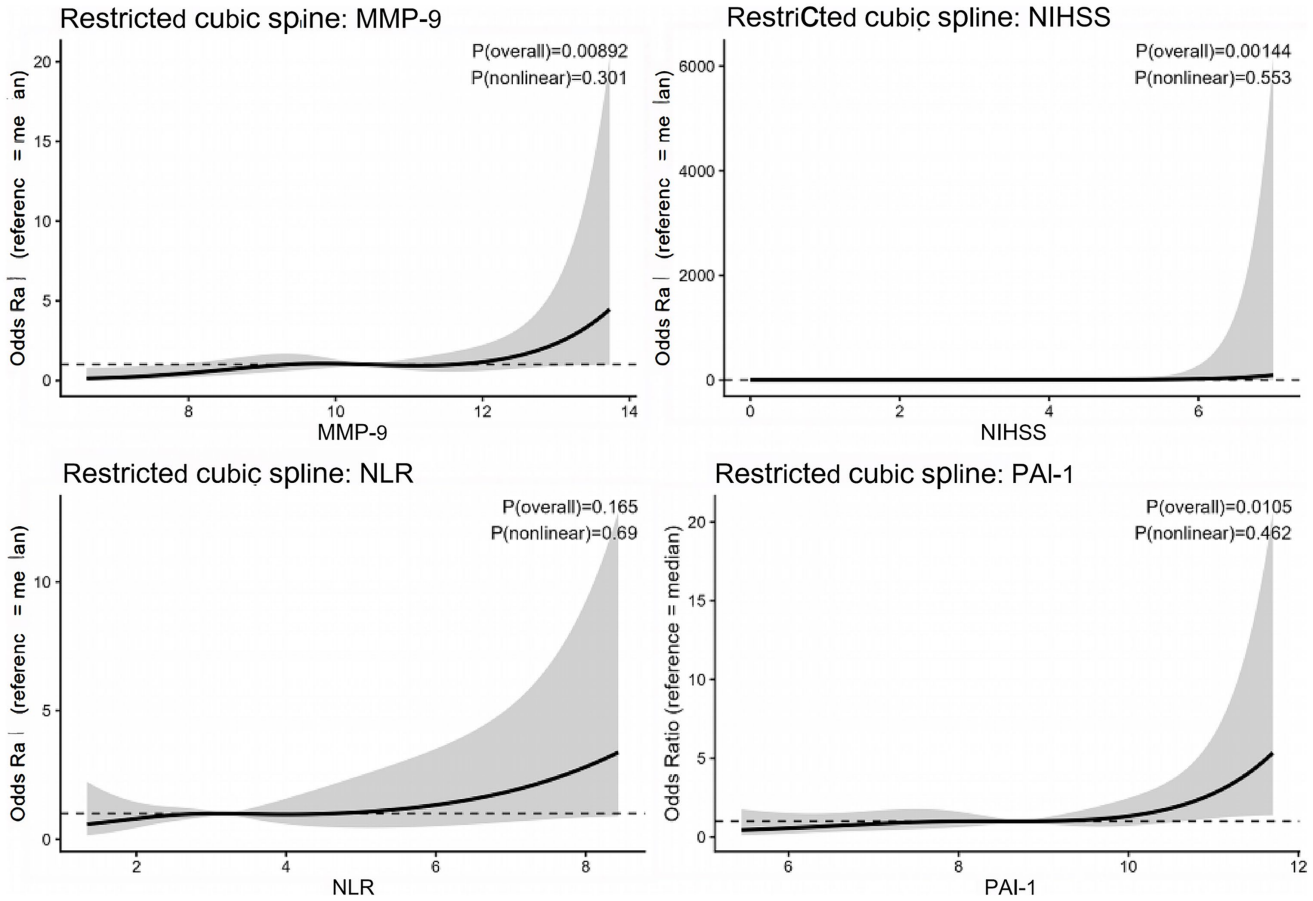
Restricted cubic spline analyses of continuous predictors and 90-day poor outcome. Restricted cubic spline models were used to evaluate the linearity between each continuous predictor and the log odds of poor outcome. The solid line represents the adjusted odds ratio across the range of each variable, and the shaded area denotes the 95% confidence interval. All predictors showed no significant evidence of nonlinearity (all *P* for nonlinearity > 0.05). NIHSS, National Institutes of Health Stroke Scale; NLR, neutrophil-to-lymphocyte ratio; PAI-1, plasminogen activator inhibitor-1; MMP-9, matrix metalloproteinase-9.

Before multivariable modeling, we assessed the linearity assumption for each continuous predictor using restricted cubic splines with four knots placed at the 5th, 35th, 65th, and 95th percentiles. As shown in Figure 2, the relationships between the log odds of poor outcome and NIHSS score, PAI-1, MMP-9, and NLR were all adequately linear, with *p* values for nonlinearity of 0.553, 0.462, 0.301, and 0.690, respectively (Supplementary Table S2). Collinearity diagnostics revealed no significant multicollinearity among these four predictors, with variance inflation factors all below 1.25 (Supplementary Table S3). These findings support the inclusion of all four variables as continuous terms in the subsequent multivariable logistic regression models.

### 3.6 Multivariable analysis identified NIHSS, MMP-9, and NLR as independent predictors, with PAI-1 retained in the final model

To identify independent predictors of 90-day poor outcome, three multivariable logistic regression models were constructed (Table 4). Model 1 included admission NIHSS score only; Model 2 included the three biomarkers (PAI-1, MMP-9, and NLR); Model 3 combined all four variables.

**Table 4:** Multivariable logistic regression models for predicting 90-day poor outcome in elderly AIS patients.

Model	Variable	*β* (SE)	OR (95% CI)	*p*
M1: NIHSS-only	Intercept	−1.99 (0.44)	—	<0.001
	NIHSS	0.62 (0.14)	1.86 (1.44–2.51)	<0.001
M2: Biomarker-only	Intercept	−8.43 (1.79)	—	<0.001
	PAI-1	0.38 (0.12)	1.46 (1.17–1.86)	0.001
	MMP-9	0.36 (0.11)	1.44 (1.17–1.81)	0.001
	NLR	0.31 (0.11)	1.36 (1.11–1.73)	0.007
M3: Combined	Intercept	−11.73 (2.54)	—	<0.001
	NIHSS	0.80 (0.19)	2.24 (1.60–3.36)	<0.001
	PAI-1	0.25 (0.14)	1.29 (0.98–1.73)	0.077
	MMP-9	0.54 (0.15)	1.71 (1.32–2.35)	<0.001
	NLR	0.35 (0.14)	1.42 (1.12–1.91)	0.011

Data are presented as regression coefficient (β), standard error (SE), odds ratio (OR), and 95% confidence interval (CI). All models used poor 90-day outcome (mRS > 2) as the dependent variable. NIHSS was modeled per 1-point increase; PAI-1 and MMP-9 per 1 ng/mL increase; NLR per 1-unit increase. The full regression equations are:

M1: logit(P) = −1.994 + 0.619 × NIHSS; M2: logit(P) = −8.431 + 0.377 × PAI-1 + 0.362 × MMP-9 + 0.305 × NLR; M3: logit(P) = −11.731 + 0.804 × NIHSS + 0.252 × PAI-1 + 0.539 × MMP-9 + 0.349 × NLR.

PAI-1 did not reach conventional statistical significance in M3 (P = 0.077) but was retained due to its consistent effect direction and contribution to overall model fit (see Supplementary Table S4 for AIC/AICc comparison, and Table 5 and Section 3.7 for discriminative performance).

AIS, acute ischemic stroke; NIHSS, National Institutes of Health Stroke Scale; PAI-1, plasminogen activator inhibitor-1; MMP-9, matrix metalloproteinase-9; NLR, neutrophil-to-lymphocyte ratio; mRS, modified Rankin Scale.

In the combined model (Model 3), higher admission NIHSS score (OR 2.24 per point, 95% CI, 1.60–3.36; *p* < 0.001), elevated MMP-9 (OR 1.71 per ng/mL, 95% CI, 1.32–2.35; *p* < 0.001), and higher NLR (OR 1.42 per unit, 95% CI, 1.12–1.91; *p* = 0.011) remained significantly associated with poor outcome. PAI-1 did not reach conventional statistical significance (OR 1.29 per ng/mL, 95% CI, 0.98–1.73; *p* = 0.077), but the direction of effect was consistent with the univariable analysis and with its hypothesized pathophysiological role. To formally evaluate whether inclusion of PAI-1 introduced overfitting, we compared models with and without PAI-1 using both the Akaike information criterion (AIC) and the small-sample corrected AIC (AICc). The combined model including PAI-1 (Model 3) yielded lower AIC (101.2 vs. 103.5) and AICc (101.7 vs. 103.9) than the reduced model without PAI-1 (ΔAIC = 2.3; Supplementary Table S4), indicating a modest improvement in model fit with inclusion of PAI-1. Bootstrap internal validation further suggested limited optimism in the final model. Together with the improved discriminative performance shown in Section 3.7 and Table 5, these findings support the retention of PAI-1 in the final model. D-dimer, which was significant in univariable analysis, was excluded after multivariable adjustment to maintain model parsimony. The full regression equations for each model are provided in the table notes.

**Table 5:** Performance comparison of the three prediction models.

Model	*n*	LR *χ*^2^ (df)	df	P (LR)	AIC	AUC (95% CI)	DeLong P*(vs M3)
M1: NIHSS-only	113	33.9 (1)	1	<0.001	125.2	0.781 (0.696–0.866)	0.003
M2: Biomarker-only	113	33.2 (3)	3	<0.001	130.0	0.791 (0.709–0.874)	0.005
M3: Combined	113	64.0 (4)	4	<0.001	101.2	0.889 (0.831–0.946)	Reference

Model definitions: M1 includes only admission NIHSS score; M2 includes PAI-1, MMP-9, and NLR; M3 includes all four variables. Likelihood ratio (LR) *χ*^2^ and Akaike information criterion (AIC) assess overall model fit. Area under the curve (AUC) values with 95% CIs were derived from ROC analysis. Pairwise AUC comparisons were performed using the DeLong test; *p* values shown are for comparisons with M3. No significant difference was observed between M1 and M2 (DeLong *P* = 0.868, not shown).

AIS, acute ischemic stroke; NIHSS, National Institutes of Health Stroke Scale; PAI-1, plasminogen activator inhibitor-1; MMP-9, matrix metalloproteinase-9; NLR, neutrophil-to-lymphocyte ratio.

**Figure 3:**
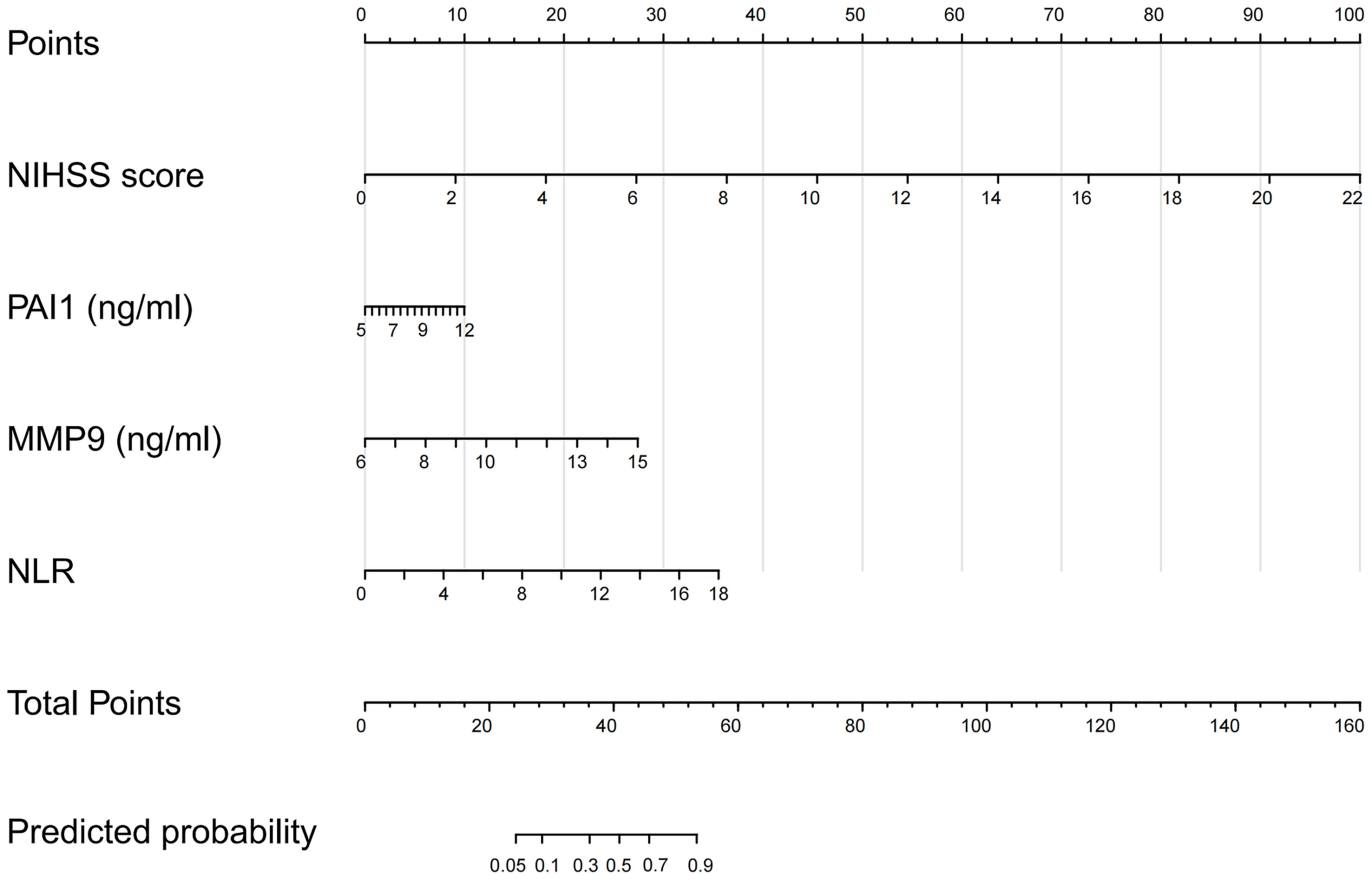
Nomogram for predicting probability of poor 90-day outcome in elderly AIS patients. Nomogram constructed based on the combined multivariable logistic regression model (Model 3, Table 4). To use the nomogram, locate the patient’s value on each variable axis, draw a vertical line upward to the “Points” scale to obtain the corresponding points for that variable. Sum the points for all four variables, locate the total on the “Total Points” axis, and draw a vertical line downward to the “Probability of Poor Outcome” axis to obtain the individual predicted risk of an unfavorable 90-day outcome (mRS > 2). Example: A patient with an admission NIHSS score of 4 (≈ 18 points), PAI-1 of 10 ng/mL (≈ 7 points), MMP-9 of 10 ng/mL (≈ 12 points), and NLR of 4 (≈ 8 points) would have a total of approximately 45 points, corresponding to an estimated 90-day poor-outcome probability of approximately 65%. NIHSS, National Institutes of Health Stroke Scale (per point); PAI-1, plasminogen activator inhibitor-1 (ng/mL); MMP-9, matrix metalloproteinase-9 (ng/mL); NLR, neutrophil-to-lymphocyte ratio (per unit). The model intercept and coefficients used for nomogram construction are provided in Table 4.

Based on Model 3, a nomogram was constructed to estimate the individual probability of 90-day poor outcome (Figure 3). In practice, clinicians can use this probability to guide individualized decisions. For example, a predicted probability exceeding 50% might prompt consideration of intensified monitoring, early rehabilitation referral, or discussion of goals of care with the family. Conversely, a probability below 20% may reassure that standard care is sufficient, avoiding unnecessary interventions. The specific threshold can be tailored to the clinical context: a lower threshold (e.g., 30%) could be used when considering low-risk interventions such as outpatient rehabilitation, whereas a higher threshold (e.g., 60%) might be appropriate for high-intensity actions like transfer to a neurointensive care unit or enrollment in an experimental trial. This flexibility accommodates varying risk tolerances and resource availability across different healthcare settings.

### 3.7 The combined model outperformed NIHSS alone in predicting poor outcome

**Figure 4:**
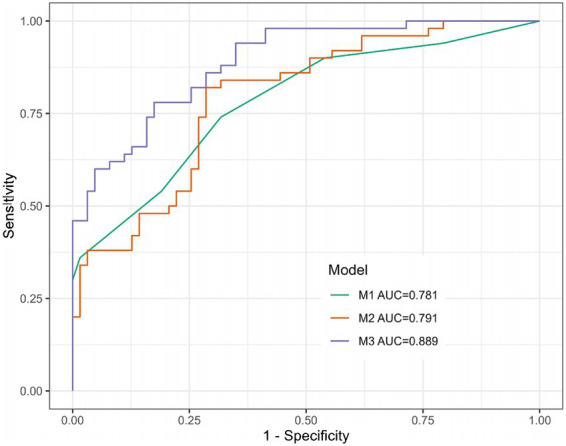
Receiver operating characteristic curves for the three prediction models. Receiver operating characteristic (ROC) curves comparing the discriminative ability of the three multivariable logistic regression models for predicting 90-day poor outcome (mRS > 2) in elderly AIS patients. The combined model (M3, blue line) includes admission NIHSS score, PAI-1, MMP-9, and NLR. The NIHSS-only model (M1, green line) includes only admission NIHSS score. The biomarker-only model (M2, red line) includes PAI-1, MMP-9, and NLR. AIS, acute ischemic stroke; NIHSS, National Institutes of Health Stroke Scale; PAI-1, plasminogen activator inhibitor-1; MMP-9, matrix metalloproteinase-9; NLR, neutrophil-to-lymphocyte ratio; mRS, modified Rankin Scale.

The discriminative performance of the three models for predicting 90-day poor outcome was assessed by ROC analysis (Figure 4) and compared using the DeLong test (Table 5). The combined model (Model 3) achieved an AUC of 0.889 (95% CI, 0.831–0.946), which was significantly higher than that of the NIHSS-only model (Model 1: AUC, 0.781, 95% CI, 0.696–0.866; DeLong *p* = 0.003) and the biomarker-only model (Model 2: AUC, 0.791, 95% CI, 0.709–0.874; *p* = 0.005). No significant difference was observed between Model 1 and Model 2 (*p* = 0.868). Likelihood ratio tests showed that all three models outperformed the null model (all *p* < 0.001). Model 3 also had the lowest AIC (101.2 vs. 125.2 for Model 1 and 130.0 for Model 2), indicating the best overall fit.

### 3.8 Model validation and calibration

**Figure 5:**
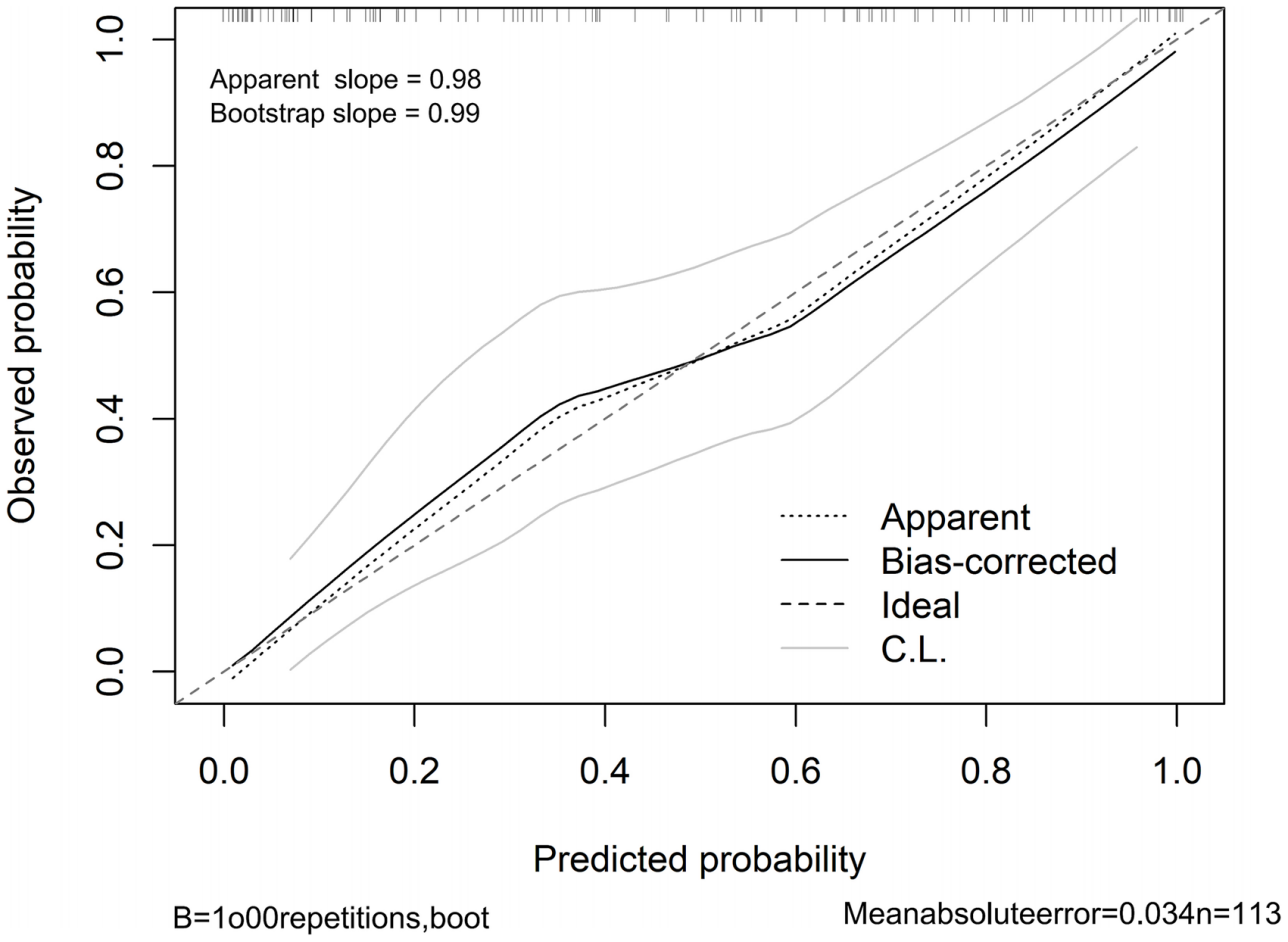
Calibration curve of the combined model for predicting 90-day poor outcome. Calibration curve assessing the agreement between predicted probabilities and observed outcomes for the combined model (M3). The *x*-axis represents the predicted probability of poor 90-day outcome (mRS > 2) from the model, and the *y*-axis represents the observed frequency of poor outcome. The solid line indicates the bias-corrected calibration curve derived from bootstrap resampling (*B* = 1,000), and the dashed diagonal line represents ideal calibration (perfect agreement). Points closer to the diagonal line indicate better calibration. AIS, acute ischemic stroke; mRS, modified Rankin Scale.

The combined model demonstrated satisfactory calibration, with the calibration curve showing close agreement between predicted probabilities and observed outcomes across the entire range of risk (Figure 5). The calibration intercept was 0.009 (95% CI, –0.105 to 0.122) and the calibration slope was 0.981 (95% CI, 0.777–1.184), indicating no evidence of systematic over- or under-estimation. After bootstrap correction, the optimism-adjusted intercept and slope were 0.006 (95% CI, –0.005 to 0.016) and 0.985 (95% CI, 0.965–1.011), respectively (Supplementary Table S5). The Brier score for the combined model was 0.135 (95% CI, 0.100–0.175), which was lower than those for the NIHSS-only model (0.182, 95% CI, 0.149–0.219) and the biomarker-only model (0.186, 95% CI, 0.150–0.220) (Supplementary Table S6), indicating superior overall prediction accuracy.

Internal validation using bootstrap resampling (*B* = 1,000) yielded an optimism-corrected C-index of 0.874 for the combined model, compared with the original apparent C-index of 0.889, corresponding to an optimism of 0.015 (Supplementary Table S7). The optimism-corrected C-indices for M1 and M2 were 0.782 and 0.773, respectively. These results indicate minimal overfitting and good internal validity of the combined model.

### 3.9 Decision curve analysis

**Figure 6:**
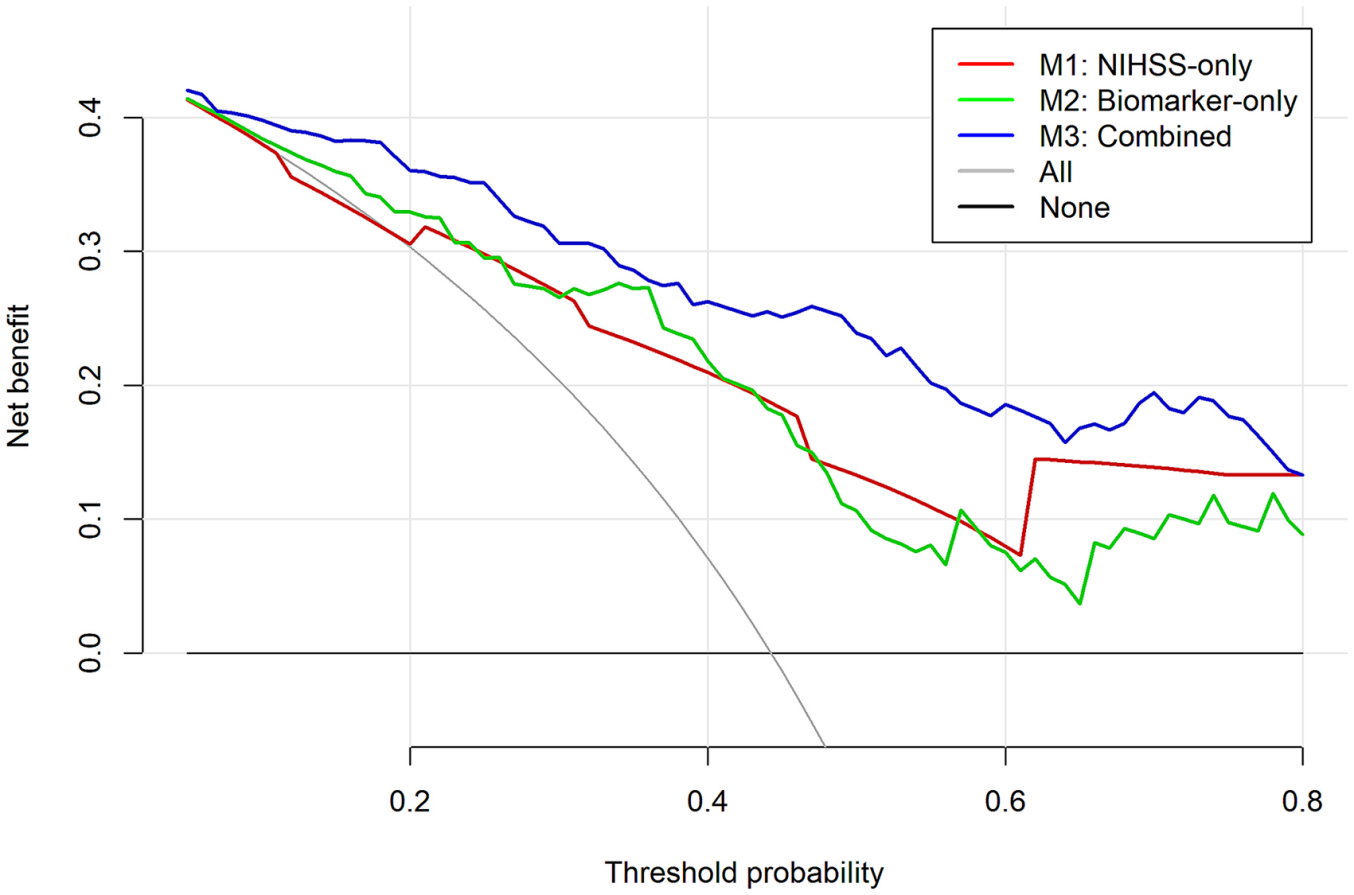
Decision curve analysis for the three prediction models. Decision curves comparing the clinical net benefit of the three models for predicting 90-day poor outcome (mRS > 2). The *y*-axis represents net benefit, and the *x*-axis represents the threshold probability at which a clinician or patient would consider intervention (e.g., intensified monitoring or early rehabilitation) worthwhile. The combined model (M3, blue line) includes NIHSS, PAI-1, MMP-9, and NLR; the NIHSS-only model (M1, red line) includes only NIHSS; the biomarker-only model (M2, green line) includes PAI-1, MMP-9, and NLR. The horizontal black line represents the “treat-none” strategy (net benefit = 0), and the gray dashed line represents the “treat-all” strategy. Models with curves farther from these reference lines provide greater net clinical benefit. AIS, acute ischemic stroke; NIHSS, National Institutes of Health Stroke Scale; PAI-1, plasminogen activator inhibitor-1; MMP-9, matrix metalloproteinase-9; NLR, neutrophil-to-lymphocyte ratio; mRS, modified Rankin Scale.

Decision curve analysis was used to assess the clinical utility of the three models (Figure 6). The combined model (M3) provided net benefit over a broad range of threshold probabilities (approximately 0.15–0.80), exceeding both the “treat-all” and “treat-none” strategies. This suggests that using M3 to guide clinical decision-making may yield better outcomes than intervening in all patients or in none.

The NIHSS-only model (M1) showed net benefit over a narrower range (approximately 0.20–0.65), whereas the biomarker-only model (M2) was beneficial only within a limited range (approximately 0.25–0.55). Overall, M3 demonstrated the greatest potential clinical usefulness across a wide spectrum of risk thresholds, supporting its value for individualized risk stratification in elderly AIS patients.

### 3.10 Results were robust to adjustment for onset-to-blood draw time

To assess whether sampling time influenced model performance, onset-to-blood draw time was added to the combined model (M3 + time). As shown in Supplementary Table S8, the coefficients and statistical significance of the original predictors (NIHSS, PAI-1, MMP-9, and NLR) remained essentially unchanged after adjustment. The AUC of M3 + time was 0.891 (vs. 0.889 for M3), the Brier score was 0.134 (vs. 0.135), and the AIC increased only slightly (102.9 vs. 101.2). Onset-to-blood draw time itself was not significantly associated with outcome (*p* = 0.600). These results indicate that the predictive performance of the combined model is robust to variation in blood-sampling timing after stroke onset.

## 4 Discussion

AIS in the elderly is associated with high morbidity and mortality, and accurate early risk stratification is essential for optimizing care (14). In this study, we investigated the prognostic value of three biomarkers representing distinct pathophysiological pathways—PAI-1 (fibrinolysis), NLR (inflammation), and MMP-9 (blood–brain barrier disruption)—in elderly patients with AIS, and developed a nomogram integrating these biomarkers with the admission NIHSS score. Our main findings are: (i) serum levels of PAI-1, NLR, and MMP-9 were significantly elevated in AIS patients compared to non-AIS controls; (ii) higher levels of these biomarkers were associated with an increased risk of poor 90-day functional outcome; (iii) a combined model incorporating NIHSS, PAI-1, NLR, and MMP-9 showed excellent discrimination (AUC, 0.889) and calibration, and significantly outperformed models based on NIHSS alone or biomarkers alone; (iv) decision curve analysis demonstrated that the combined model provides net clinical benefit over a wide range of threshold probabilities (0.15–0.80). These findings suggest that adding these three pathway-specific biomarkers to the NIHSS score may improve early risk prediction in elderly AIS patients managed without reperfusion therapy—a population that remains substantial in clinical practice, particularly in settings where timely access to thrombolysis or thrombectomy is limited.

The pathophysiological rationale for selecting PAI-1, NLR, and MMP-9 is firmly grounded in established stroke mechanisms. PAI-1, the principal inhibitor of tissue-type plasminogen activator (tPA), profoundly impairs fibrinolysis by stabilizing thrombi, thereby promoting persistent ischemia and extending infarct size; elevated PAI-1 levels have been consistently linked to worse functional outcomes and hemorrhagic transformation post-stroke (15, 16). NLR serves as an accessible composite marker of systemic inflammation and stroke-induced immunosuppression: neutrophilia drives neuroinflammation via cytokine release and reactive oxygen species, while lymphopenia reflects T-cell apoptosis and impaired immune surveillance, both exacerbating secondary brain injury (17, 18). MMP-9, a gelatinase that degrades tight junction proteins and extracellular matrix in the neurovascular unit, compromises blood–brain barrier integrity, facilitating vasogenic edema, hemorrhage, and long-term neurological deficits (19, 20).

These biomarkers target complementary, interconnected domains of AIS pathophysiology—fibrinolysis/thrombosis (PAI-1), inflammation/immunity (NLR), and blood–brain barrier disruption (MMP-9)—which collectively underpin the multifaceted ischemic cascade. No single biomarker can encapsulate this complexity, and multi-marker panels have emerged as superior for prognostication by capturing additive or synergistic risks (21, 22). Recent studies reinforce this: multi-inflammatory indices like NLR combined with systemic inflammation response index (SIRI) provide incremental prognostic value beyond single markers in AIS cohorts (23); nomogram models incorporating NLR alongside clinical variables outperform NIHSS alone for short-term outcomes (24); and MMP-9 panels enhance prediction of barrier-related complications when integrated with inflammatory metrics (20). Systematic evidence further highlights that biomarker combinations from thrombosis, inflammation, and barrier domains consistently yield higher AUCs than monomarkers (25).

In our study, this multi-domain strategy translated into tangible gains: the biomarker-only model (M2) matched NIHSS discrimination (AUC, 0.791 vs. 0.781), while the combined model (M3) significantly outperformed both (AUC, 0.889; DeLong *p* < 0.01 vs. M1/M2), with superior calibration, Brier score, and decision curve net benefit. These findings affirm the incremental value of our panel, offering a more nuanced risk profile than any individual component.

Although PAI-1 did not reach conventional statistical significance in the multivariable model (*p* = 0.077), its inclusion was retained because it improved overall model fit and calibration, and the direction of effect was consistent with its biological role. This underscores that even markers with borderline significance can contribute meaningfully to multi-marker panels when supported by mechanistic rationale (21). Moreover, the fact that the biomarker-only model achieved discrimination comparable to NIHSS suggests that these three blood markers collectively capture prognostic information similar to a detailed neurological examination. Importantly, adding them to NIHSS yielded the best performance, demonstrating that combining clinical assessment with pathway-specific biomarkers provides incremental discriminative ability (4, 15). This is clinically relevant because PAI-1, NLR, and MMP-9 can be measured rapidly from a single fasting blood sample obtained on the first morning after admission, potentially enabling early in-hospital risk stratification. This is particularly relevant for non-reperfused elderly patients, in whom early identification of high-risk individuals may guide decisions regarding intensified monitoring, prioritized referral to higher-level stroke centers, and timely initiation of rehabilitation planning (23).

We did not propose a fixed cutoff for defining “high risk” because threshold selection depends on the clinical context and the risk–benefit balance of subsequent interventions. Instead, we provide a nomogram that yields a continuous probability estimate, allowing individualized decision-making (see section 3.6 for practical examples). Decision curve analysis confirmed that the combined model offers net clinical benefit across a wide range of threshold probabilities (0.15–0.80), supporting its potential utility in guiding management decisions.

This study has several limitations: First, it is a single-center prospective study (*n* = 113) with a modest sample size. Although bootstrap internal validation has been performed on the model, its generalizability requires external validation through multi-center, prospective, large-sample cohort studies. Second, exclusion of patients who received intravenous thrombolysis or mechanical thrombectomy was a prespecified design decision to minimize treatment-related heterogeneity and confounding by indication. While this may enhance internal validity by reducing treatment-related heterogeneity, it substantially restricts external generalizability: the model should not be directly extrapolated to reperfused populations, in whom the relationship between baseline biomarkers and outcome may be modified by recanalization success. Prospective multicenter studies that include both reperfused and non-reperfused cohorts with treatment-stratified analyses are planned as a next step. Third, biomarkers were measured at a single standardized time point (fasting, first morning after admission) rather than serially. Although this protocol ensures specimen homogeneity and is practical for routine clinical use, dynamic changes over the first 72 h post-stroke might provide additional prognostic information (26, 27), Fourth, all AIS patients received the same standardized acute management immediately after admission (statins, antiplatelet therapy, and hydration). Thus, any medication-related influence on biomarkers was uniform across patients and does not introduce bias; however, this unavoidable timing is acknowledged as a limitation. Finally, the lack of an external validation cohort means our performance estimates may be optimistic; large, multicenter prospective studies with serial biomarker measurements are needed to confirm and enhance the model’s utility.

## 5 Conclusion

This study demonstrates that a nomogram integrating NIHSS with PAI-1, MMP-9, and NLR provides useful prediction of 90-day functional outcome in non-reperfused elderly patients with AIS, with good discrimination, calibration, and potential clinical utility. Among these biomarkers, MMP-9 and NLR showed independent associations with outcome, while PAI-1 contributed to overall model performance despite borderline statistical significance. These findings support the potential value of this multi-pathway biomarker panel for early in-hospital risk stratification in non-reperfused elderly AIS patients. Future research should validate and optimize the model through large-scale prospective cohorts and explore the integration of dynamic monitoring and multi-omics information, ultimately promoting more individualized and precise prognostic management in elderly AIS.

## Data Availability

The raw data supporting the conclusions of this article will be made available by the authors, without undue reservation.
