# Supplementary material for: PAI-1, MMP-9, and NLR combined with NIHSS for predicting 90-day poor functional outcome in elderly acute ischemic stroke: a prospective observational cohort study
Source: Front Neurol. 2026 Apr 15;17:1793227. doi: 10.3389/fneur.2026.1793227 (PMC13124988; doi:10.3389/fneur.2026.1793227)
Supplement: Supplementary file 1 [file Table_1.DOCX]

****Supplementary Table S1. Multivariate linear regression analysis of biomarker levels comparing AIS patients versus controls****

| **Biomarker** | **Adjusted Mean Difference(β)** | **95% CI** | **P value** |
| --- | --- | --- | --- |
| PAI‑1 (ng/mL) | 2.537 | 1.848 – 3.226 | < 0.001 |
| MMP‑9 (ng/mL) | 1.413 | 0.662 – 2.165 | < 0.001 |
| NLR | 1.502 | 0.722 – 2.282 | < 0.001 |

****Table Note:****
Models were adjusted for age, sex, smoking, drinking, diabetes, and hypertension.

The β coefficient represents the adjusted mean difference in biomarker levels for AIS patients compared with controls (reference group). Variance inflation factors (VIF) for all covariates were below 2 (range: 1.03–1.52), indicating no multicollinearity. Adjusted R² values were 0.263 for PAI‑1, 0.112 for MMP‑9, and 0.052 for NLR.
