# Supplementary material for: PAI-1, MMP-9, and NLR combined with NIHSS for predicting 90-day poor functional outcome in elderly acute ischemic stroke: a prospective observational cohort study
Source: Front Neurol. 2026 Apr 15;17:1793227. doi: 10.3389/fneur.2026.1793227 (PMC13124988; doi:10.3389/fneur.2026.1793227)
Supplement: Supplementary file 2 [file Table_2.DOCX]

### ****Supplementary Table S2. Restricted cubic spline analysis for nonlinearity assessment****

| **Variable** | **Knots placement** | **P for overall association** | **P for nonlinearity** |
| --- | --- | --- | --- |
| NIHSS | 5th, 35th, 65th, 95th | 0.001 | 0.553 |
| PAI-1 | 5th, 35th, 65th, 95th | 0.011 | 0.462 |
| MMP-9 | 5th, 35th, 65th, 95th | 0.009 | 0.301 |
| NLR | 5th, 35th, 65th, 95th | 0.165 | 0.690 |

Table Note:

Each variable was modeled using restricted cubic splines with four knots, adjusted for the other three variables as linear terms. P values were derived from Wald tests for the overall association (testing whether the spline coefficients were jointly zero) and for nonlinearity (testing whether the coefficient of the nonlinear spline terms were zero). A P value for nonlinearity > 0.05 indicates that the linear assumption is adequate.
