# Supplementary material for: PAI-1, MMP-9, and NLR combined with NIHSS for predicting 90-day poor functional outcome in elderly acute ischemic stroke: a prospective observational cohort study
Source: Front Neurol. 2026 Apr 15;17:1793227. doi: 10.3389/fneur.2026.1793227 (PMC13124988; doi:10.3389/fneur.2026.1793227)
Supplement: Supplementary file 3 [file Table_3.DOCX]

### ****Supplementary Table S3. Variance inflation factor for assessment of multicollinearity****

| **Variable** | **VIF** |
| --- | --- |
| NIHSS | 1.24 |
| PAI-1 | 1.11 |
| MMP-9 | 1.23 |
| NLR | 1.17 |

Table Note:

Variance inflation factor (VIF) was calculated from a linear regression model including the four predictors. A VIF < 5 is generally considered to indicate no significant multicollinearity. The low VIF values (< 1.25) observed here suggest negligible collinearity among the predictors, supporting their simultaneous inclusion in the multivariable model.
