# Supplementary material for: PAI-1, MMP-9, and NLR combined with NIHSS for predicting 90-day poor functional outcome in elderly acute ischemic stroke: a prospective observational cohort study
Source: Front Neurol. 2026 Apr 15;17:1793227. doi: 10.3389/fneur.2026.1793227 (PMC13124988; doi:10.3389/fneur.2026.1793227)
Supplement: Supplementary file 4 [file Table_4.DOCX]

****Supplementary Table S4. Model fit comparison with and without PAI‑1****

| **Model** | **Variables** | **AIC** | **AICc** | **ΔAIC** |
| --- | --- | --- | --- | --- |
| Model 3 | NIHSS + MMP‑9 + NLR + PAI‑1 | 101.2 | 101.7 | reference |
| Model 4 | NIHSS + MMP‑9 + NLR | 103.5 | 103.9 | **2.3** |

****Table Note:****
Models were constructed using multivariable logistic regression with 90-day poor outcome (mRS > 2) as the dependent variable. AIC, Akaike Information Criterion; AICc, small-sample corrected AIC. ΔAIC represents the difference in AIC relative to Model 3 (reference; the model with the lowest AIC). A ΔAIC > 2 is generally considered to indicate meaningfully poorer model fit. The inclusion of PAI-1 in Model 3 reduced both AIC and AICc compared with Model 4, supporting improved model fit without evidence of overfitting.

Abbreviations: NIHSS, National Institutes of Health Stroke Scale; PAI-1, plasminogen activator inhibitor-1; MMP-9, matrix metalloproteinase-9; NLR, neutrophil-to-lymphocyte ratio; mRS, modified Rankin Scale.
