# Supplementary material for: PAI-1, MMP-9, and NLR combined with NIHSS for predicting 90-day poor functional outcome in elderly acute ischemic stroke: a prospective observational cohort study
Source: Front Neurol. 2026 Apr 15;17:1793227. doi: 10.3389/fneur.2026.1793227 (PMC13124988; doi:10.3389/fneur.2026.1793227)
Supplement: Supplementary file 5 [file Table_5.DOCX]

### ****Supplementary Table S5****

| ****Metric**** | ****Estimate**** | ****95% CI**** |
| --- | --- | --- |
| ****Apparent calibration intercept**** | 0.009 | –0.105 to 0.122 |
| ****Apparent calibration slope**** | 0.981 | 0.777 to 1.184 |
| ****Bootstrap‑corrected intercept**** | 0.006 | –0.005 to 0.016 |
| ****Bootstrap‑corrected slope**** | 0.985 | 0.965 to 1.011 |
| ****Apparent Brier score**** | 0.135 | – |
| ****Bootstrap‑corrected Brier score**** | 0.127 | 0.092 to 0.164 |

****Table note:**** Calibration intercept and slope were derived from a logistic regression model with the linear predictor as the only covariate. Bootstrap estimates are based on 1000 resamples with optimism correction. The Brier score ranges from 0 to 1, with lower values indicating better overall prediction accuracy. For the combined model (M3: NIHSS + PAI‑1 + MMP‑9 + NLR).
