# Supplementary material for: PAI-1, MMP-9, and NLR combined with NIHSS for predicting 90-day poor functional outcome in elderly acute ischemic stroke: a prospective observational cohort study
Source: Front Neurol. 2026 Apr 15;17:1793227. doi: 10.3389/fneur.2026.1793227 (PMC13124988; doi:10.3389/fneur.2026.1793227)
Supplement: Supplementary file 6 [file Table_6.DOCX]

### ****Supplementary Table S6. Brier scores for the three prediction models****

| **Model** | **Brier score (95% CI)** |
| --- | --- |
| M1: NIHSS-only | 0.182 (0.149–0.219) |
| M2: Biomarker-only | 0.186 (0.150–0.220) |
| M3: Combined | 0.135 (0.100–0.175) |

The Brier score measures overall prediction accuracy, ranging from 0 to 1, with lower values indicating better accuracy. Values were calculated from logistic regression predictions and 95% confidence intervals were obtained via bootstrap resampling (B = 1000). Model definitions: M1 includes only admission NIHSS score; M2 includes PAI‑1, MMP‑9, and NLR; M3 includes all four variables.

AIS, acute ischemic stroke; NIHSS, National Institutes of Health Stroke Scale; PAI‑1, plasminogen activator inhibitor‑1; MMP‑9, matrix metalloproteinase‑9; NLR, neutrophil‑to‑lymphocyte ratio.
