# Supplementary material for: PAI-1, MMP-9, and NLR combined with NIHSS for predicting 90-day poor functional outcome in elderly acute ischemic stroke: a prospective observational cohort study
Source: Front Neurol. 2026 Apr 15;17:1793227. doi: 10.3389/fneur.2026.1793227 (PMC13124988; doi:10.3389/fneur.2026.1793227)
Supplement: Supplementary file 7 [file Table_7.DOCX]

### ****Supplementary Table S7. Bootstrap internal validation of the three models****

| **Model** | **Original C-index** | **Optimism-corrected C-index** | **Optimism** |
| --- | --- | --- | --- |
| M1: NIHSS-only | 0.781 | 0.782 | –0.001 |
| M2: Biomarker-only | 0.791 | 0.773 | 0.019 |
| M3: Combined | 0.889 | 0.874 | 0.015 |

**Table Note**

Internal validation was performed using bootstrap resampling with 1000 iterations. The optimism‑corrected C‑index provides an estimate of model performance adjusted for overfitting, with smaller optimism values indicating less overfitting. Model definitions: M1 includes only admission NIHSS score; M2 includes PAI‑1, MMP‑9, and NLR; M3 includes all four variables.

AIS, acute ischemic stroke; NIHSS, National Institutes of Health Stroke Scale; PAI‑1, plasminogen activator inhibitor‑1; MMP‑9, matrix metalloproteinase‑9; NLR, neutrophil‑to‑lymphocyte ratio.
