# Supplementary material for: PAI-1, MMP-9, and NLR combined with NIHSS for predicting 90-day poor functional outcome in elderly acute ischemic stroke: a prospective observational cohort study
Source: Front Neurol. 2026 Apr 15;17:1793227. doi: 10.3389/fneur.2026.1793227 (PMC13124988; doi:10.3389/fneur.2026.1793227)
Supplement: Supplementary file 8 [file Table_8.DOCX]

### ****Supplementary Table S 8. Sensitivity analysis: effect of adjusting for onset‑to‑blood draw time****

| **variables** | **M3（β±SE）** | **P** | **M3+**onset‑to‑blood draw time**（β±SE）** | **P** |
| --- | --- | --- | --- | --- |
| NIHSS | 0.8041 ± 0.1869 | <0.001 | 0.8051 ± 0.1883 | <0.001 |
| NLR | 0.3492 ± 0.1381 | 0.011 | 0.3477 ± 0.1369 | 0.011 |
| PAI‑1 | 0.2522 ± 0.1425 | 0.077 | 0.2583 ± 0.1436 | 0.072 |
| MMP9 | 0.5388 ± 0.1459 | <0.001 | 0.5594 ± 0.1534 | <0.001 |
| onset‑to‑blood draw time | — | — | 0.0117 ± 0.0223 | 0.600 |

****Model performance metrics****

| **index** | **M3** | **M3+**onset‑to‑blood draw time**** | **variety** |
| --- | --- | --- | --- |
| AUC | 0.889 | 0.891 | +0.002 |
| Brier | 0.135 | 0.134 | −0.001 |
| AIC | 101.18 | 102.90 | +1.72 |

**Table Note:**

Model 3 (M3) includes admission NIHSS, PAI‑1, MMP‑9, and NLR. M3+time includes the same four variables plus onset‑to‑blood draw time (hours). Coefficients (β) and standard errors (SE) are from logistic regression models with 90‑day poor outcome as the dependent variable. AUC, area under the ROC curve; AIC, Akaike information criterion. The small changes in coefficients and performance metrics indicate that the model’s predictive ability is robust to adjustment for sampling time.

AIS, acute ischemic stroke; NIHSS, National Institutes of Health Stroke Scale; PAI‑1, plasminogen activator inhibitor‑1; MMP‑9, matrix metalloproteinase‑9; NLR, neutrophil‑to‑lymphocyte ratio.
